# DNA methylation and mRNA expression of *SYN III*, a candidate gene for schizophrenia

**DOI:** 10.1186/1471-2350-9-115

**Published:** 2008-12-22

**Authors:** Brenda C Murphy, Richard L O'Reilly, Shiva M Singh

**Affiliations:** 1 Molecular Genetics Unit, Department of Biology and Division of Medical Genetics The University of Western Ontario, London, Ontario N6A 5B7, Canada

## Abstract

**Background:**

The synapsin III (*SYN III*) gene on chromosome 22q is a candidate gene for schizophrenia susceptibility due to its chromosome location, neurological function, expression patterns and functional polymorphisms.

**Methods:**

This research has established the mRNA expression of *SYN III *in 22 adult human brain regions as well as the methylation specificity in the closest CpG island of this gene. The methylation specificity studied in 31 brain regions (from a single individual) was also assessed in 51 human blood samples (representing 20 people affected with schizophrenia and 31 normal controls) including a pair of monozygotic twin discordant for schizophrenia and 2 non-human primates.

**Results:**

The results show that the cytosine methylation in this genomic region is 1) restricted to cytosines in CpG dinucleotides 2) similar in brain regions and blood and 3) appears conserved in primate evolution. Two cytosines (cytosine 8 and 20) localized as the CpG dinucleotide are partially methylated in all brain regions studied. The methylation of these sites in schizophrenia and control blood samples was variable. While cytosine 8 was partially methylated in all samples, the distribution of partial to complete methylation at the cytosine 20 was 22:9 in controls as compared to 18:2 in schizophrenia (p = 0.82). Also, there is no difference in methylation between the affected and unaffected member of a monozygotic twin pair.

**Conclusion:**

The variation in *SYN III *methylation studied is 1) not related to schizophrenia in the population sample or a monozygotic twin pair discordant for schizophrenia and 2) not related to the mRNA level of *SYN IIIa *in different human brain regions.

## Background

*Synapsins *(*SYN*) encode for neuron-specific phosphoproteins which associates with the cytoplasmic surface of synaptic vesicles. They play critical roles in neurotransmitter release, synaptogenesis and clustering of synaptic vesicles at active zones and function as modulators of synaptic strength by acting at both pre- and postdocking levels [1]. They secure synaptic vesicles to actin filaments controlling the number of vesicles available for release at the nerve terminus. They play a broad role during neuronal development, including the formation and maintenance of synaptic contacts among central neurons. In humans distinct genes for *SYN I *(Xp11.4-p11.2), *SYN II *(3p) and *SYN III *(22q12-q13) give rise to 10 distinct isoforms [2,3] and each synapsin has a specific role during the elongation of undifferentiated processes and their posterior differentiation into axons and dendrites [4]. The primary protein domains A, C and E are conserved among all major SYN isoforms and *SYN *III has a novel domain J [2,3]. The isoforms of *SYN *III can be divided into neural (IIIa to IIId) and nonneural (IIIe and IIIf) based on their expression [3]. Further, although isoforms IIIa to IIIc are present in fetal and adult brain, IIIa is clearly the predominant isoform expressed in adult brain [3]. Isoform IIId is only expressed in fetal brain [3]. The gene encoding *SYN III *is a candidate for schizophrenia susceptibility as polymorphisms in the promoter and coding regions of *SYN III *have been implicated in schizophrenia [5-12]. Also, expression studies have identified some differences in *SYN *in patients with schizophrenia [13-16]. Such results although encouraging does not offer an unequivocal support for a definitive role of *SYN III *in this heterogeneous and common disease. They suggest that any role for *SYN III *in schizophrenia, if present, is not obvious.

It is important to note that schizophrenia (SZ) is a complex disorder. Evidence of genetic risk factor(s) in schizophrenia is based on family and adoption studies [17,18]. Concordance for schizophrenia in monozygotic twins (MZ) is reported to be ~41–86% as compared to 16% in dizygotic twins [19-23]. The reduced concordance (<100%) for this disorder in MZ twins suggests that not all schizophrenia determinants involve gene mutations [24]. Also, a number of non-genetic risk factors in the causation of schizophrenia have been recognized that include malnutrition, dietary folate, viral infections, birth order, obstetric complications and other random events [25-29]. Also, epigenetic factors, particularly DNA methylation of CpG islands in promoter and enhancer regions of transcriptionally active genes have been proposed for the etiology of schizophrenia [30-36] as alterations in the methylation pattern may alter the level of mRNA and/or protein [37-39]. Some of the potential loci that may be affected by methylation in schizophrenia include SOX 10 [40], COMT [35,36] and DRD2 [41], but the direct role of the gene specific methylation in schizophrenia has not been established. In a recent study involving epigenetic profiling of promoters Mills et al. [42] found evidence for psychosis-associated DNA-methylation differences in numerous loci, including several involved in glutamatergic and GABAergic neurotransmission, brain development, and other processes functionally linked to schizophrenia and bipolar disorder. This phenomenon has the potential to be a promising area of research in schizophrenia. This phenomenon has been hypothesized and reported in a number of human diseases [43] and may account for discordance of monozygotic twins [44]. This hypothesis is particularly attractive in schizophrenia, since searching for traditional genetic mutations have not yielded reproducible results.

Since *SYN III *remains an attractive candidate gene for schizophrenia, this study assesses the expression of *SYN IIIa *(the predominant isoform in adult human brain) and cytosine DNA methylation of *SYN III's *closest CpG island. The results provide a detailed evaluation of the expression of *SYN *IIIa, as well as the pattern of cytosine methylation in *SYN III's *distal CpG island in 31 human brain regions from one human male. Also, included are methylation studies on the blood genomic DNA from 20 schizophrenia patients and 31 unaffected controls and a pair of monozygotic twin discordant for this disease. The results identify cell type specific brain *SYN III *mRNA expression differences, and two CpG sites that are differently methylated in the brain and blood samples. These results are compared to those obtained from non-human primates.

## Methods

### Source of DNA and RNA

This research was undertaken following an approval from the Ethics Review Board of the University of Western Ontario. This report is based on the analysis of 82 human and 2 non-human primate DNA samples. The 82 human DNA samples represent 31 different human brain regions (from one individual) and 51 blood samples (20 schizophrenia patients [SZ1–SZ20] and 31 un affected controls [C1–C31]) from Southwestern Ontario, Canada. Each patient and control was assessed for signs and symptoms of schizophrenia using DSM IV. All diagnosis was made by Richard L. O'Reilly (Clinical Psychiatrist). The 31 brain samples representing specific brain regions were obtained from Ambion (Austin, TX) who coordinated a custom autopsy on a 63-year old Caucasian male that died of liver cancer. No information was available on the patient's treatment and medication but the donor's serology results were negative for HIV, Syphilis, HBV and HTLV. The 2 non-human primate (cynomologous monkey and baboon) blood samples were obtained from the animal care facility of the University of Western Ontario.

DNA was extracted from fresh blood using QIAamp DNA blood Maxi Kit (Qiagen; Valencia, CA) while RNA was extracted using Trizol (Invitrogen; Carlsbad, CA) following the manufacturer's instructions. DNA from 31 frozen brain tissue was extracted using DNAzol (Molecular Research Centre; Cincinnati, OH). Only 22 of the 31 specific brain regions yielded RNA using TRIzol (Invitrogen; Carlsbad, CA). cDNA synthesis from total RNA was made using Superscript II (Invitrogen; Carlsbad, CA) and purified using QIAquick PCR purification kit (Qiagen; Valencia, CA).

### *SYN III's *distal CpG island

**Figure 1:**
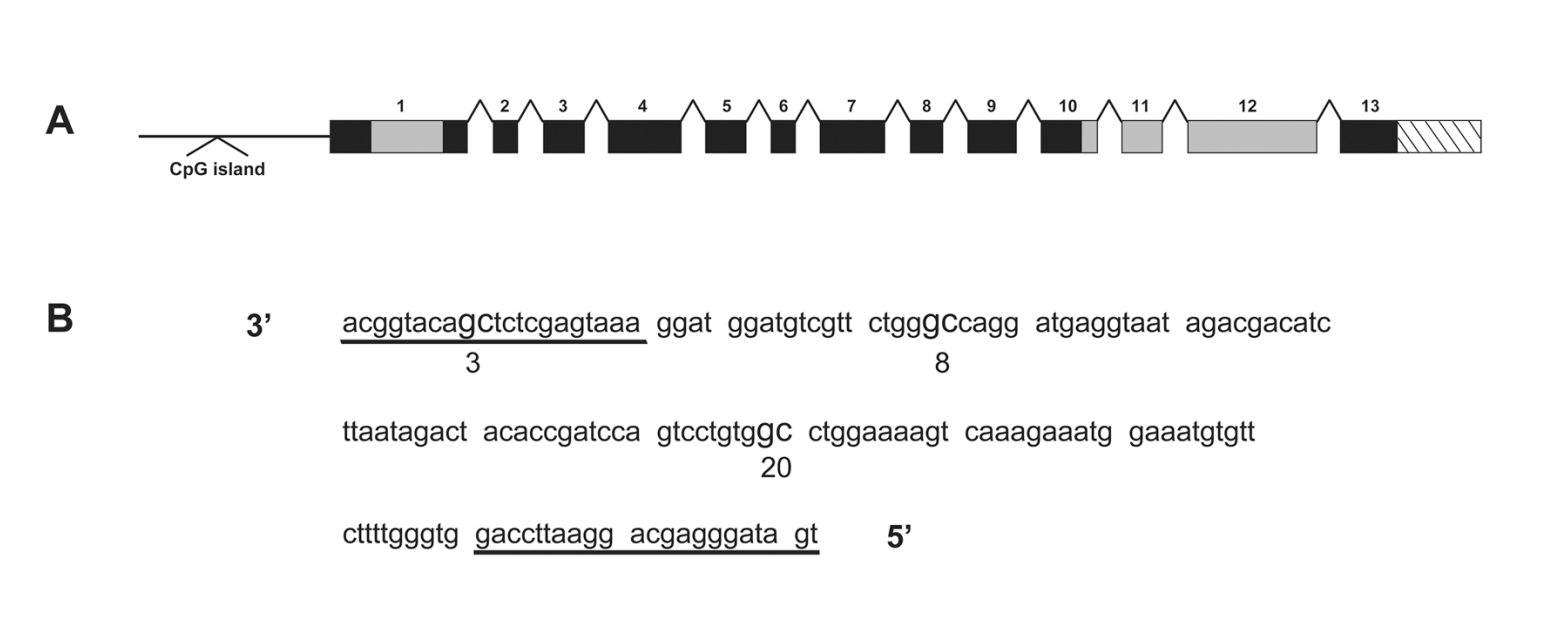
**Genomic organization of SYN III gene and its distal CpG island on chromosome 22q12 (A) and the nucleotide sequence (antisense strand of NCBI Accession Number Z83846, nucleotides 87366 to 87522) used for methylation analysis (B)**. Underlined sequences represent the location of sodium bisulfite PCR primers, cytosines are numbered 1–26 with three 5'CpGs 3' (#3, 8 and 20) shown in bold.

The *SYN III *sequence (Accession number Z83846) was subjected to annotation for gene organization that included exons, introns and CpG island. The closest CpG island is located ~50 kb upstream from *SYN III *ATG site in a putative *SYN III *enhancer region (Figure 1A). A portion of this region formed the focus of methylation studies using modification of genomic DNA by sodium bisulfite. PCR primers were developed for sodium bisulfite modified genomic DNA (Figure 1B) for use in amplification and sequencing of this region from all samples.

### Sodium Bisulfite Conversion of gDNA

We modified a previously established method [45]. Briefly, 2 ug of each DNA sample was cut overnight with *Hind III *(Invitrogen; Carlsbad, CA) at 37°C. Completely digested gDNA was phenol/chloroformed and reprecipitated with 3 M sodium acetate (pH 5.1) and 100% ethanol. The pellet was vacuum dried and re-suspended in 40 uL of sterile water. The purified *Hind III *digested DNA was exposed to sodium bisulfite, which in theory converts unmethylated cytosine to uracil while methylated cytosines remain unaltered. The DNA was purified with a Wizard DNA Clean-up System (Promega; Madison, WI) following the manufacture's protocol. Desalted samples were reprecipitated with 3 M sodium acetate (pH 5.1) and 100% ethanol. The pellet was vacuum dried and resuspended in 100 uL of sterile water.

### PCR amplification of sodium bisulfite treated gDNA

Two primers compatible to the sodium bisulfite converted sequence of the distal CpG island (Figure 1B) were optimized in a 25 uL PCR reaction to amplify a 157 bp fragment. Each sample was PCR amplified. The 25 uL aliquot was run on a 6% PAG, stained with ethidium bromide and photographed using an Alpha Innotech Gel Doc System.

### Sequencing of sodium bisulfite amplified PCR

The PCR band was excised, crushed, suspended in water and DNA eluted overnight at 37°C. The polyacrylamide was separated from the DNA aqueous using a Costar Spin X column (45 um filter). DNA was precipitated with 3 M sodium acetate (pH 5.1) and 100% ethanol. The pellet was vacuum dried and resuspended in 20 uL of water. Two 5 uL aliquots of this DNA were each diluted in 9 uL of sterile water and 5 uL (20 pmoles/uL) of the Fwd or Rev primer. These 19 uL solutions were sent for sequencing at the London Regional Genomics Sequencing Facility of the University of Western Ontario, London Ontario. PCR products were directly sequenced using dGTP chemistry and an Applied Biosystems (Foster City, CA) 377 ABI Automatic Sequencer. The resulting fluorograms allowed the identification of methylation for each cytosine in the sequence. As a further confirmation of the sequence of the sodium bisulfite PCR product was undertaken on the monozygotic twin pair, SZ19 (affected with schizophrenia) and C2 (not affected) following cloning in Promega's PGEM-T Easy Vector (Madison, WI). Six clones from each individual were sequenced to confirm the results obtained by direct sequencing of the PCR products.

### Semi-quantitative RT-PCR using cDNA (see above)

Primers for *Actin *(5'acaatgagctgcgtgtggct3' and 5'tctccttaatgtacagcacg3'), *GAPD *(5'tgaaggtcggagtcaacggatttggt3'and 5'catgtgggccatgaggtccaccac3') and *SYN *IIIa (5'agcatctccatccatccacagcc3' and 5'cagagatggaggttctcatgtaagcc3') were each optimized separately in 25 uL PCR reaction (Invitrogen, Carlsbad, CA) to amplify 360, 983 and 958 bp fragments respectively. Each sample was PCR amplified (linear phase was empirically determined to be 25 cycles), and the 25 uL aliquot was run on a 6% PAG, stained with ethidium bromide, photographed and quantitated using an Alpha Innotech Gel Doc System. These RT-PCRs were repeated three times i.e. first with the original cDNA, repeated with the same cDNA and repeated with fresh cDNA (made from the original mRNA).

## Results

### Expression of *SYN IIIa *in human brain

**Figure 2:**
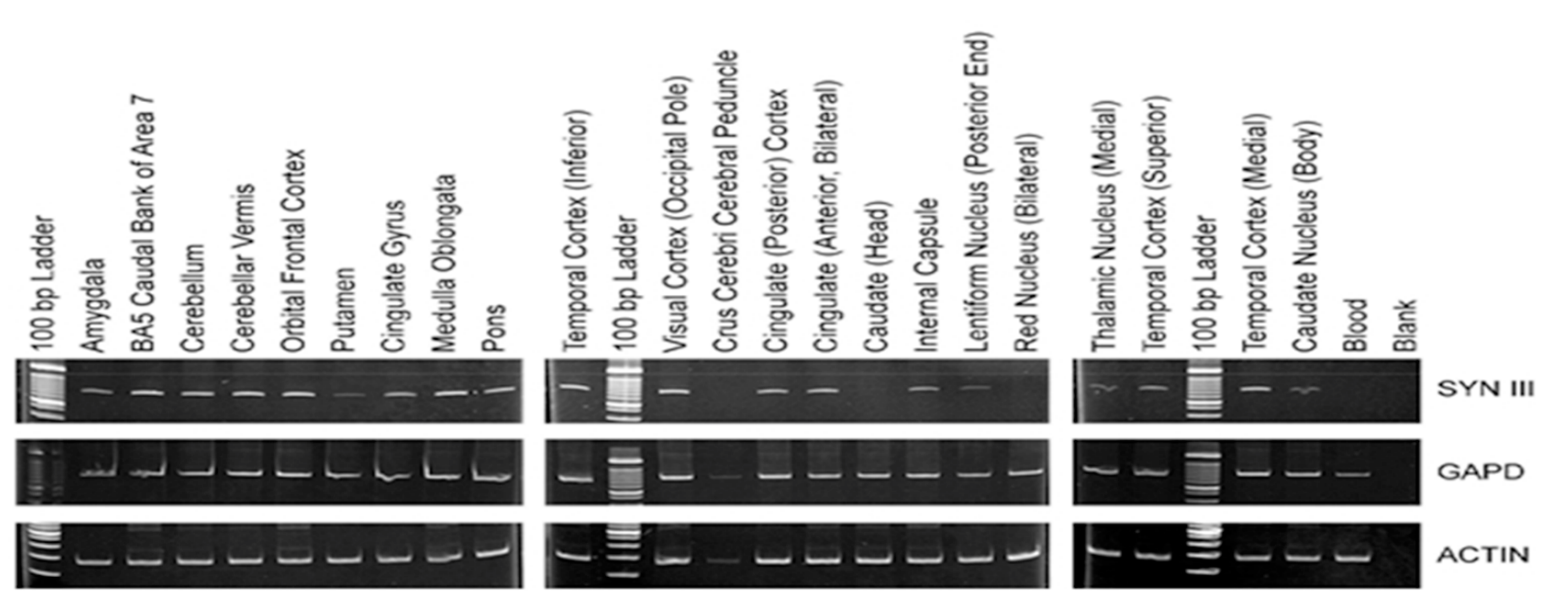
**RT-PCR results for *SYN III *and two internal controls (*GAPD *and *Actin*) in 22 human brain-specific regions and blood**.

*SYN IIIa *mRNA quantitation in 22 brain regions for which the RNA samples were obtained was based on three experiments (original cDNA, repeat of orginal cDNA and fresh cDNA from original RNA) involving semi-quantitative RT-PCR (Figure 2). The expression of *SYN IIIa *in each case was assessed in relation to two housekeeping genes (*GAPD *and *Actin*), that were used as internal controls. The results show that the *SYN IIIa *was expressed in most brain regions examined. However, the level of expression was different in different regions of the human brain. Relatively higher expression was seen in the cortex (temporal, visual and cingulated), cerebellum and BA5 caudal bank while lower expression was seen in the amygdala, pons, internal capsule and temporal cortex (superior). Further, the expression of this mRNA was very low or absent in putamen, lentiform nucleus, red nucleus, thalamic nucleus, caudate nucleus (body), crus cerebri cerebral and caudate (head and body). The results establish that SYN IIIa expression is variable in the human brain regions. In comparison, the *SYN IIIa *mRNA was not detected in the blood sample, under the sensitivity of the methodology used.

### Cytosine methylation in *SYN *III's distal CpG island in the human brain

**Figure 3:**
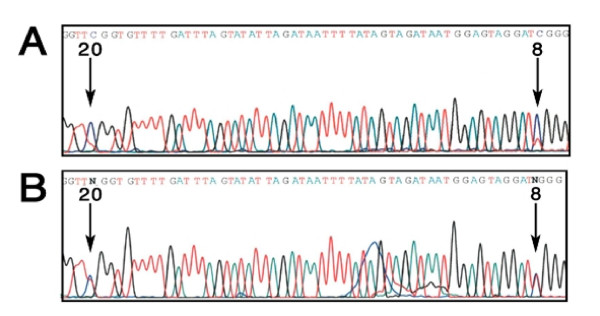
**Sequence flurograms (using reverse primer) show partial methylation of cytosine 8 and 20 in human brain specific regions**. A. (cerebellar vermis) shows prominent methylation of cytosine 8 and 20 (both sites showing C and a trace of T), B. (caudate; head) shows partial methylation (N call by sequencer) of cytosines 8 and 20.

Figure 1 shows the genomic organization of the human *SYN III *gene and DNA sequence of a portion of the distal CpG island used for studies on cytosine methylation. This region contains 26 cytosines that are numbered 1 to 26 in a 3' to 5' direction towards the reverse primer. The direct sequencing of the PCR product of sodium bisulfite modified gDNA on ABI sequencer has allowed us to assess the fluorograms as called by the sequencer and further appraised by visual observation. Here, a comparison of sodium bisulfite modified gDNA sequence with the NCBI sequence accession number Z83846 show G, A or T nucleotides at all the expected sites. Theoretically, the methylated cytosines are maintained at their original positions while unmethylated cytosines have been converted to T. Also, all cytosines in CpG dinucleotides are unaffected by the sodium bisulfite and cytosines in CpN (where N is not guanine) were completely converted. Note, the unmethylated cytosines in the sequence being investigated serve as controls that the sodium bisulfite pretreatment is working appropriately. Overall, the results suggest that only cytosines in the CpG dinucleotides are subject to methylation in this region of the genome. A close examination of the fluorograms revealed that one of the 3 CpG (cytosine 8) was partially methylated in all DNA samples that included regions of brain and the blood. This partial methylation was read as an N by the sequencer or showed a C as well as T (or G and trace of A on the complementary strand) peaks to different degrees. Table 1 summarizes the methylation specificity of the cytosines at the CpG dinucleotides in the 31 brain specific regions. The degree of cytosine methylation at these CpG dinucleotides was estimated to range from ~50 to 80% (Figure 3). What is also apparent is that the degree of methylation of CpG dinucleotides in this genomic region is specific to different regions of the brain. Given that the brains regions examined were derived from a single individual, the results may or may not be representative for all brains.

**Table 1:** Specific methylation at Cytosine 8 & 20 in 31 human brain regions.

31 Brain Regions (originating from one individual) and Blood	mRNA expression	Methylation status of the Cytosines in 3 CpG dinucleotides (see Figure 1 for cytosine numbering)		
		**Cytosine Number**		
		3	8	20

Amygdala	Medium	●	○/●	○/●

BA5 Caudal Bank of Area 7	High	●	○/●	○/●

Corpus Callosum	NA	●	○/●	○/●

Cerebellum	High	●	○/●	○/●

Cerebellar Vermis	High	●	○/●	○/●

Hippocampus	NA	NA	○/●	○/●

Orbital Frontal Cortex	High	●	○/●	○/●

Putamen	Very low/absent	●	○/●	○/●

Temporal Lobe	NA	●	○/●	○/●

Thalamic Nucleus (Lateral)	NA	NA	○/●	○/●

White Cortical Matter	NA	NA	○/●	○/●

Cingulate Gyrus	Medium	●	○/●	○/●

Gray Cortical Matter	NA	●	○/●	○/●

Medulla Oblongata	High	●	○/●	○/●

Pons	Medium	●	○/●	○/●

Temporal Cortex (Inferior)	High	●	○/●	○/●

Visual Cortex (Occipital Pole)	High	●	○/●	○/●

Crus Cerebri Cerebral Peduncle	Very low/absent	●	○/●	○/●

Cingulate (Posterior Cortex)	High	●	○/●	○/●

Cingulate (Anterior Bilateral)	High	●	○/●	○/●

Cingulate (Anterior Cortex)	NA	●	○/●	○/●

Caudate (Head)	Very low/absent	●	○/●	○/●

Internal Capsule	Medium	●	○/●	○/●

Lentiform Nucleus (Posterior End)	Very low/absent	●	○/●	○/●

Red Nucleus (Bilateral)	Very low/absent	●	○/●	○/●

Thalamic Nucleus (Medial)	Very low/absent	●	○/●	○/●

Temporal Cortex (Superior)	Medium	●	○/●	○/●

Temporal Cortex (Medial)	High	●	○/●	○/●

Caudate Nucleus (Body)	Very low/absent	●	○/●	○/●

Internal Capsule (Posterior Limb)	NA	●	○/●	○/●

Thalamus (Lateral Geniculate Nucleus)	NA	●	○/●	○/●

Blood (from table 1)	NA	●	○/●	○/●or ●

● methylated, ○ unmethylated, ○/● partial methylation and NA not available.

### SYN IIIa methylation in schizophrenia patients

**Figure 4:**
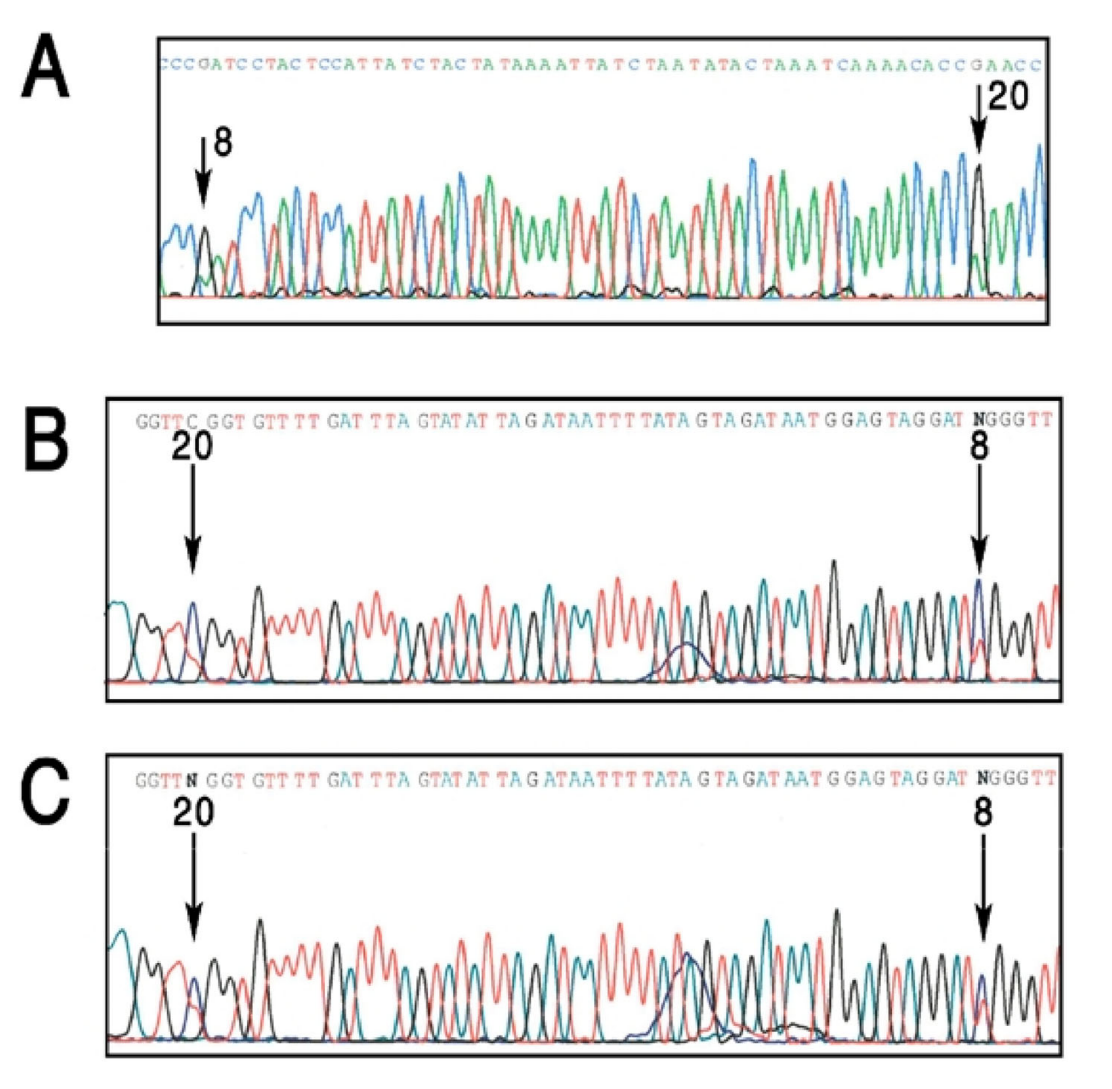
**Sequence flurograms (Panel A using forward primer; C and D using reverse primer)**. Panel A show partial methylation (G and trace of A) of cytosine 8 and 20. Panel B show partial methylation (N call by sequencer) of cytosine 8 and partial methylation (G and trace of A) of cytosine 20. Panel C show partial methylation (N call by sequencer) at both cytosine 8 and 20. Panel A, B and C are the results of individuals labeled SZ2, C3 and C11 respectively.

**Figure 5:**
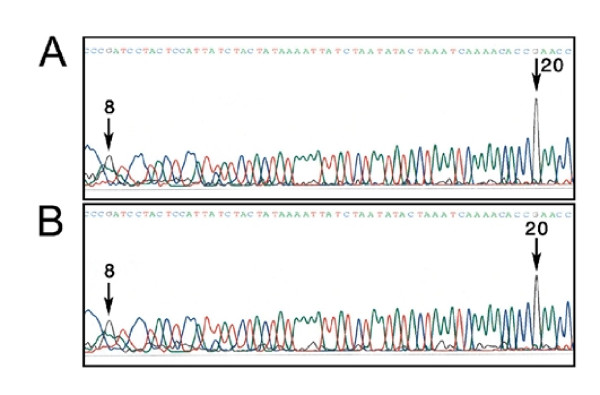
**Methylation property of cytosine 8 and 20 in two non-human primates; cynomologous monkey (A) and baboon (B)**.

Methylation patterns in the proximal CpG island of *SYN IIIa *of DNA from blood samples from patients with schizophrenia and normal controls were similar to those generated from human brain as cytosine methylation was restricted only to the CpG sites, only. The methylation pattern at cytosine 8 in 20 patients and 31 controls (Table 2, Figure 4) was comparable but not identical. Here the methylation of the cytosine 8 was similar in the blood and brain but unlike the brain, cytosine 20 in blood samples was either partially (~50–80%) or completely methylated. The distribution of the cytosine 20 partial (22 controls and 18 schizophrenia patients) vs complete (9 controls and 2 schizophrenia patients) methylation was not different (Table 2, χ^2 ^= 3.14, p = 0.08) in this sample from Southwestern Ontario. Further the methylation results on the monozygotic co-twins discordant for schizophrenia was not different. Additional familial results also suggested that the pattern of methylation for cytosine 8 and 20 is compatible with familial inheritance. Interestingly, the sequence of this genomic region is conserved in non-human primates and its methylation in the two non-human primates (cynomologous monkey and baboon) is similar to the human blood (Figure 5).

**Table 2:** Methylation of SYN III promoter cytosine 8 and 20 in 51 blood samples representing controls and patients with schizophrenia.

**Unaffected Control Number**	**Methylation of cytosine number 8 and 20**	
	**8**	**20**
C1	○/●	○/●

C2	○/●	○/●

C3	○/●	○/●

C4	○/●	○/●

C5	○/●	○/●

C6	○/●	○/●

C7	○/●	○/●

C8	○/●	●

C9	○/●	●

C10	○/●	○/●

C11	○/●	○/●

C12	○/●	○/●

C13	○/●	●

C14	○/●	○/●

C15	○/●	●

C16	○/●	○/●

C17	○/●	○/●

C18	○/●	○/●

C19	○/●	○/●

C20	○/●	●

C21	○/●	●

C22	○/●	●

C23	○/●	○/●

C24	○/●	●

C25	○/●	○/●

C26	○/●	○/●

C27	○/●	●

C28	○/●	○/●

C29	○/●	○/●

C30	○/●	○/●

C31	○/●	○/●

**Schizophrenia Patient Number**	**Methylation of cytosine number 8 and 20**	

	**8**	**20**

SZ1	○/●	○/●

SZ2	○/●	○/●

SZ3	○/●	○/●

SZ4	○/●	○/●

SZ5	○/●	○/●

SZ6	○/●	○/●

SZ7	○/●	○/●

SZ8	○/●	○/●

SZ9	○/●	○/●

SZ10	○/●	○/●

SZ11	○/●	○/●

SZ12	○/●	○/●

SZ13	○/●	○/●

SZ14	○/●	○/●

SZ15	○/●	○/●

SZ16	○/●	●

SZ17	○/●	○/●

SZ18	○/●	○/●

SZ19	○/●	●

SZ20	○/●	○/●

**Totals for complete versus partial methylation of cytosine 20**		

	**Controls**	**Patients**

Complete Methylation	9	2

Partial Methylation	22	18

## Discussion

The SYN III gene has remained a putative candidate for schizophrenia, by virtue of its function [1,4], expression [3,13-16], linkage and association [5-12] in humans and synaptic trafficking alterations associated with knockout mice for Syn I and Syn II [46]. Overall, the published reports on association and co-segregation of DNA polymorphism and schizophrenia although indicative, are not without exceptions and a definitive role for *SYN III *in this complex and heterogeneous disease, remains to be established. Because epidemiological studies of schizophrenia fail to support candidate gene mutation of Mendelian origin, we hypothesized that epigenetic mechanisms (i.e., cytosine hypermethylation of CpG islands present in the promoter of this genes) may partake in the down-regulation of this gene in patients with schizophrenia. This study reports novel results on the neuronal expression of *SYN III*, methylation specificity associated with its distal CpG island and its implications in schizophrenia and expression of this gene in the human brain.

### Expression and cytosine methylation of *SYN IIIa *in the human brain

The results included in this report support previous reports citing *SYN III *and in particular *SYN IIIa *expression in adult brain [2,3]. Further, it establishes that the *SYN IIIa *expression in the brain is region and tissue-type specific. The question of how this variability is achieved is of interest, which must be assessed in relevant cell-types. Determinants of differential *SYN III *expression may influence or determine a normal expression of this gene. Any abnormality in such determinants particularly CpG methylation in *SYN III*'s CpG island may cause *SYN *IIIa associated abnormalities.

The *SYN III *cytosines examined show that cytosine methylation is restricted to CpG dinucleotides only. This pattern is seen in all tissues and cell-types analyzed (i.e. 31 brain regions, genomic DNA from human blood and blood DNA from two nonhuman primates). Such results argue that the observed pattern of methylation is an inherent and conserved feature of this region of the human genome. Within this region, cytosine 8 is partially methylated while cytosine 20 is either partially or fully methylated in human brain (Table 1), human blood (Table 2) and blood samples from nonhuman primates. Of interest is the observation on partial methylation. In addition, the results argue that in absence of prohibitive brain tissues, readily available blood DNA may serve as a source for such studies. Similar methylation in human and non-human primate blood DNA in this sequence conserved region may suggest that this methylation is evolutionarily relevant and could be biologically significant.

Also, the individual specific partial/full methylation at the cytosine 20 site across individuals may imply epigenetic polymorphism in the population. In this context the nature and source of this variability remains hypothetical and may represent an aspect of parent of origin effect. The relevance of this polymorphism in human brains could not be assessed as the 31 brain regions assessed were derived from a single individual. More important, since methylated and unmethylated cytosine 20 was observed in DNA from patients with schizophrenia as well as the controls and this distribution was not significantly different between the two groups, it was ruled out as being involved in schizophrenia. This conclusion is also supported by the results on monozygotic twin pair discordant for schizophrenia (Figure 5). These and other results included further argue that this epigenetic polymorphism is a genetic property of this DNA sequence that is selected and maintained across generations and during phylogeny. Interestingly, the twin pair studied in this research was 51 years of age. The identical methylation at these sites in twin pairs may suggest that this site is not subject to aging effects [44]. In conclusion, the results included in this report offer support for variation in the level of CpG methylation in this part of the *SYN IIIa *gene promoter, however it is not involve in the development of schizophrenia or the variable expression of this gene in the brain regions. Future experiments are required to explain tissue specific *SYN III *mRNA expression differences.

## Pre-publication history

The pre-publication history for this paper can be accessed here:



## References

[B1] Evergren E, Benfenati F, Shupliakov O (2007). The synapsin cycle: A view from the synaptic endocytic zone. J Neurosci Res.

[B2] Kao H, Porton B, Czernik AJ, Feng J, Yiu G, Haring M, Benfenati F, Greengard P (1998). A third member of the synapsin gene family. Proc Natl Acad Sci USA.

[B3] Porton B, Kao H, Greengard P (1999). Characterization of transcripts from the synapsin III gene locus. J of Neurochem.

[B4] Ferreira A, Rapoport M (2002). The synapsins: beyond the regulation of neurotransmitter release. Cell Mol Life Sci.

[B5] Stober G, Meyer J, Nanda I, Wienker TF, Saar K, Knapp M, Jatzke S, Schmid M, Lesch KP, Beckmann H (2000). Linkage and family-based association study of schizophrenia and the synapsin III locus that maps to chromosome 22q13. Am J Med Genet.

[B6] Ohtsuki T, Ichiki R, Toru M, Arinami T (2000). Mutational analysis of the synapsin III gene on chromosome 22q12-q13 in schizophrenia. Psychiatry Res.

[B7] Ohmori O, Shinkai T, Hori H, Kojima H, Nakamura J (2000). Synapsin III gene polymorphisms and schizophrenia. Neuroscience Letters.

[B8] Imai K, Harada S, Kawanish Y, Tachikawa H, Okubo T, Susuki T (2001). Polymorphisms in the promoter an coding regions of the synapsin III gene. Neurospychobiology.

[B9] Tsai MT, Hung CC, Tsai CY, Liu MY, Su YC, Chen YH, Hsiaso KJ, Chen CH (2002). Mutation analysis of synapsin III gene in schizophrenia. Am J Med Genet.

[B10] Porton B, Ferreira A, DeLisi LE, Kao HT (2004). A rare polymorphism affects a mitogen-activated protein kinase site in synapsin III: possible relationship to schizophrenia. Biol Psychiatry.

[B11] Lachman HM, Stopkova P, Rafael MA, Saito T (2005). Association of schizophrenia in African Americans to polymorphism in synapsin III gene. Psychiatr Genet.

[B12] Lachman HM, Stopkova P, Papolos DF, Pedrosa E, Margolis B, Aghalar MR, Saito T (2006). Analysis of synapsin III-196 promoter mutation in schizophrenia and bipolar disorder. Neuropsychobiology.

[B13] Vawter MP, Thatcher L, Usen N, Hyde TM, Kleinman JE, Freed WJ (2002). Reduction of synapsin in the hippocampus of patients with bipolar disorder and schizophrenia. Mol Psychiatry.

[B14] Mirnics K, Middleton FA, Marquez A, Lewis DA, Levitt P (2000). Molecular characterization of schizophrenia viewed by microarray analysis of gene expression in prefrontal cortex. Neuron.

[B15] Halim ND, Weickert CS, McClintock BW, Hyde TM, Weinberger DR, Kleinman JE, Lipska BK (2003). Presynaptic proteins in the prefrontal cortex of patients with schizophrenia and rats with abnormal prefrontal development. Mol Psychiatry.

[B16] Porton B, Wetsel WC (2007). Reduction of synapsin III in the prefrontal cortex of individuals with schizophrenia. Schizophr Res.

[B17] Hallmayer J (2000). The epidemiology of the genetic liability of schizophrenia. Aust NZ J Psyc.

[B18] Tienari PJ, Wynne LC (1994). Adoption studies of schizophrenia. Ann Med.

[B19] Cardno AG, Marshall EJ, Coid B, Macdonald AM, Ribchester TR, Davies NJ, Venturi P, Jones LA, Lewis SW, Sham PC, Gottesman II, Farmer AE, McGuffin P, Reveley AM, Murray RM (1999). Heritability estimates for psychotic disorders: the Maudsley twin psychosis series. Arch Gen Psychiatry.

[B20] Fischer M (1973). Genetic and environmental factors in schizophrenia: a study of schizophrenic twins and their families. Acta Psychiatr Scand Suppl.

[B21] Franzek E, Beckmann H (1998). Different genetic background of schizophrenia spectrum psychosis: A twin study. Am J Psychiatry.

[B22] Kringlen E, Cramer G (1989). Offspring of Monozygotic twins discordant for schizophrenia. Arch Gen Psychiatry.

[B23] Sherman SL, DeFries JC, Gottesman II, Loehlin JC, Meyer JM, Pelias MZ, Rice J, Waldman I (1997). Recent developments in human behavioural genetics: past accomplishments and future directions. Am J Hum Genet.

[B24] O'Reilly RL, Singh SM (1996). Retroviruses and schizophrenia revisited. Am J Med Genet.

[B25] Mednick SA, Machon RA, Huttunen MO, Bonett D (1988). Adult schizophrenia following prenatal exposure to an influenza epidemic. Arch. Gen Psych.

[B26] Karlsson H, Bachmann S, Schroder J, McArthur J, Torrey EF, Yolken RH (2001). Retroviral RNA identified in the cerebrospinal fluids and brains of individuals with schizophrenia. Proc Natl Acad Sci USA.

[B27] Torrey EF, Miller J, Rawlings R, Yolken RH (1997). Seasonality of births in schizophrenia and bipolar disorder: a review of the literature. Schizophre Res.

[B28] Jablensky A (2000). Epidemiology of schizophrenia: the global burden of disease and disability. Eur Arch Psych Clin Neurosci.

[B29] Pedersen CB, Mortensen PB (2001). Evidence of a dose-response relationship between urbanicity during upbringing and schizophrenia risk. Arch Gen Psych.

[B30] Singh SM, Murphy B, O'Reilly R (2002). Epigenetic contributors to the discordance of monozygotic twins. Clin Genet.

[B31] Singh SM, Murphy B, O'Reilly R (2002). Monozygotic twins with chromosome 22q11 deletion and discordant phenotypes: updates with an epigenetic hypothesis. J Med Genet.

[B32] Singh SM, Murphy BC, O'Reilly R (2003). Involvement of gene-diet/drug interaction in DNA methylation and its contribution to complex diseases: from cancer to schizophrenia. Clin Genet.

[B33] Singh SM, McDonald P, Murphy BC, O'Reilly R (2004). Incidental neurodevelopmental episodes in the etiology of schizophrenia: An expanded model involving epigenetics and development. Clin Genet.

[B34] Petronis A (2001). The origin of schizophrenia: Genetic thesis, epigenetic antithesis, and resolving synthesis. Biol Psychiatry.

[B35] Murphy BC, O'Reilly RL, Singh SM (2005). Site-specific methylation in S-COMT promoter in 31 brain regions with implications for studies involving schizophrenia. Am J Med Gen Part B (Neuropsychiatric Genet).

[B36] Abdolmaleky HM, Cheng KH, Faraone SV, Wilcox M, Glatt SJ, Gao F, Smith CL, Shafa R, Aeali B, Carnevale J, Pan H, Papageorgis P, Ponte JF, Sivaraman V, Tsuang MT, Thiagalingam S (2006). Hypomethylation of MB-COMT promoter is a major risk factor for schizophrenia and bipolar disorder. Hum Mol Genet.

[B37] Santos F, Dean W (2004). Epigenetic reprogramming during early development in mammals. Reproduction.

[B38] Lehmann U, Brakensiek K, Kreipe H (2004). Role of epigenetic changes in hematological malignancies. Ann Hematol.

[B39] Pompeia C, Hodge DR, Plass C, Wu YZ, Marquex VE, Kelley JA, Farrar WL (2004). Microarray analysis of epigenetic silencing of gene expression in the KAS-6/1 multiple myeloma cell line. Cancer Res.

[B40] Iwamoto K, Bando M, Yamada K, Takao H, Iwayama-Shigano Y, Yoshikawa T, Kato T (2005). DNA methylation status of SOX10 correlates with its down regulation and oligodendrocyte dysfunction in schizophrenia. J Neurosci.

[B41] Petronis A, Gottesman II, Kan P, Kennedy JL, Basile VS, Paterson AD, Popendikyte V (2003). Monozygotic twins exhibit numerous epigenetic differences: clues to twin discordance?. Schizophr Bull.

[B42] Mills J, Tang T, Kaminsky Z, Khare T, Yazdanpanah S, Bouchard L, Jia P, Assadzadeh A, Flanagan J, Schumacher A, Wang SC, Petronis A (2008). Epigenomic profiling reveals DNA methylation changes associated with major psychosis. Am J Hum Genet.

[B43] Feinberg AP, Tycko B (2004). The history of cancer epigenetics. Nat Rev Cancer.

[B44] Fraga MF, Ballestar E, Paz MF, Ropero S, Setien F, Ballestar ML, Heine-Suner D, Cigudosa JC, Urioste M, Benitez J, Boiz-Chornet M, Sanchez-Qguilera A, Ling C, Carlsson E, Poulsen P, Vaag A, Stephan Z, Spector TD, Wu YZ, Plass C, Esteller M (2005). Epigenetic differences arise during the lifetime of monozygotic twins. Proc Natl Acad Sci USA.

[B45] Frommer M, McDonald LE, Millar DS, Collis CM, Watt F, Grigg GW, Molloy PL, Paul CL (1992). A genomic sequencing protocol that yields a positive display of 5-methylcytosine residues in individual DNA strands. Proc Natl Acad Sci USA.

[B46] Feng J, Chi P, Blanpied TA, Xu Y, Magarinos AM, Ferreira A, Takahashi RH, Kao H, McEwen BS, Ryan TA, Augustine GJ, Greengard P (2002). Regulation of neurotransmitter release by synapsin III. J Neurosci.

